# Determining the applicability of the RSNA radiology lexicon (RadLex) in high-grade glioma MRI reporting—a preliminary study on 20 consecutive cases with newly diagnosed glioblastoma

**DOI:** 10.1186/s12880-022-00776-8

**Published:** 2022-03-24

**Authors:** Torge Huckhagel, Christine Stadelmann, Tammam Abboud, Christian Riedel

**Affiliations:** 1 Department of Neuroradiology, University Medical Center Göttingen, Göttingen, Germany; 2 Department of Neuropathology, University Medical Center Göttingen, Göttingen, Germany; 3 Department of Neurosurgery, University Medical Center Göttingen, Göttingen, Germany

**Keywords:** Glioblastoma, Glioma, Magnetic resonance imaging, Radiology lexicon, RadLex, Radiology report

## Abstract

**Background:**

The implementation of a collective terminology in radiological reporting such as the RSNA radiological lexicon (RadLex) yields many benefits including unambiguous communication of findings, improved education, and fostering data mining for research purposes. While some fields in general radiology have already been evaluated so far, this is the first exploratory approach to assess the applicability of the RadLex terminology to glioblastoma (GBM) MRI reporting.

**Methods:**

Preoperative brain MRI reports of 20 consecutive patients with newly diagnosed GBM (mean age 68.4 ± 10.8 years; 12 males) between January and October 2010 were retrospectively identified. All terms related to the tumor as well as their frequencies of mention were extracted from the MRI reports by two independent neuroradiologists. Every item was subsequently analyzed with respect to an equivalent RadLex representation and classified into one of four groups as follows: 1. verbatim RadLex entity, 2. synonymous/multiple equivalent(s), 3. combination of RadLex concepts, or 4. no RadLex equivalent. Additionally, verbatim entities were categorized using the hierarchical RadLex Tree Browser.

**Results:**

A total of 160 radiological terms were gathered. 123/160 (76.9%) items showed literal RadLex equivalents, 9/160 (5.6%) items had synonymous (non-verbatim) or multiple counterparts, 21/160 (13.1%) items were represented by means of a combination of concepts, and 7/160 (4.4%) entities could not eventually be transferred adequately into the RadLex ontology.

**Conclusions:**

Our results suggest a sufficient term coverage of the RadLex terminology for GBM MRI reporting. If applied extensively, it may improve communication of radiological findings and facilitate data mining for large-scale research purposes.

**Supplementary Information:**

The online version contains supplementary material available at 10.1186/s12880-022-00776-8.

## Background

Currently, neuroradiological findings are generally reported by each radiologist using his or her individual vocabulary, which makes sufficient comparability of follow-up examinations by different raters as well as possible data aggregation for research purposes extremely problematic. On the other hand, there are numerous substantial benefits resulting from implementing a common language in radiology reporting. A common terminology allows for unambiguous interdisciplinary communication of findings, education of radiology residents, quality improvement, and research facilitation including data mining with the help of large-scale databases [1, 2]. Therefore, the Radiological Society of North America (RSNA) supported by the American College of Radiology, the College of American Pathologists, the National Institute of Biomedical Imaging and Bioengineering (NIBIB), the cancer Biomedical Informatics Grid (caBIG) as well as numerous other professional organizations established a comprehensive radiological lexicon termed RadLex that aims to cover the whole field of medical imaging comprising of all different radiological subspecialties [3, 4]. Even though the title RadLex implies that it might only be a conventional lexicon defining specific radiological items, in fact it should rather be regarded as an ontology which does not only contain standardized concepts and definitions. Additionally, it provides extensive background information on their specific relationships [3, 5]. A few years after the release of the first version of this radiological terminology in English language in 2005, a translation into German language was published that already comprised of more than 6,000 items [6]. Since then, both the English and German editions of the RadLex have continuously been updated and augmented and the current version 4.0 of this radiology domain lexicon already contained more than 46,000 entities at the time of publication in January, 2019 [7]. RadLex has demonstrated the most excellent results compared to other developed vocabularies in indexing radiological content collected from peer-reviewed biomedical publications, where nearly all images could be annotated with one or even multiple RadLex terms [8]. Unfortunately, due to its manual top-down construction by experts in the field, the RadLex ontology remains, by its very nature, incomplete with a large body of empirical literature revealing gaps of coverage in certain radiological fields such as mammography and chest computed tomography [9–11]. Focusing on the neurooncological domain of the neuroradiology subspecialty, to date there is no information on the applicability, performance and coverage of the RadLex vocabulary with respect to glioblastoma (GBM) magnetic resonance imaging (MRI) findings, which are known to be the most common malignant primary brain tumors (World Health Organization classification of tumors of the central nervous system grade IV) of astrocytic origin with an increased incidence with age [12]. On these grounds, we have aimed here for the first time to determine the manual transferability of free-text German-speaking MRI reports on newly diagnosed GBM cases into RadLex terminology.

## Methods

### Ethics

Prior to data collection, the study protocol was formally approved by the Institutional Review Board of the University Medical Center Göttingen (registration number: 8/8/20). All steps of the course of this investigation are in line with the Declaration of Helsinki adopted by the World Medical Association General Assembly in 1964 and its later amendments [13].

### Patients and procedures

After formal study protocol approval, 20 consecutive preoperative MRI reports of patients with newly diagnosed primary GBM between January 2010 and October 2010 were retrospectively identified by means of a comprehensive neurooncological database provided by the local department of neurosurgery. Due to the exploratory design of this feasibility study and the considerable resources required for manual extraction and assignment of technical terms, the number of cases analyzed was limited. The definite confirmation of GBM diagnosis (19X primary GBM, 1X primary gliosarcoma) was established by our colleagues of the department of neuropathology in all cases. A gliosarcoma is a rare biphasic glial and sarcomatous variant of GBM with similar prognosis following standard treatment [14]. Of note, gliosarcoma is indistinguishable from GBM when the diagnosis is only based on clinical and neuroimaging characteristics [15]. Pathological diagnoses of all included grade 4 astrocytic tumors (GBM, gliosarcoma) were based on the well-established criteria set out in the contemporary fourth edition of the World Health Organization classification of tumors of the central nervous system [16]. All preoperative brain MRI imaging was performed at our tertiary care neuroradiology center using a 3.0 Tesla MRI scanner (TrioTim; Siemens, Erlangen, Germany). Images were acquired in accordance with the latest European Society of Neuroradiology (ESNR) recommendations for glioma imaging developed from the protocol jointly published by the European Organization for Research and Treatment of Cancer (EORTC) and the United States National Brain Tumor Society (NBTS), which include at least isotropic 3D T1 weighted images before and after contrast agent application, axial 2D 3-directional diffusion weighted imaging, axial 2D T2-weighted turbo spin-echo sequences, axial 2D T2-weighted fluid attenuated inversion recovery sequences, and dynamic susceptibility contrast MR perfusion [17, 18]. Presurgical MRI scans of all patients were independently assessed by two neuroradiologists. Following the imaging procedure, all patients underwent near-term cranial neurosurgery within the next days to weeks (mean 4.4 ± 5.1 days).

### Data collection and processing

**Fig. 1:**
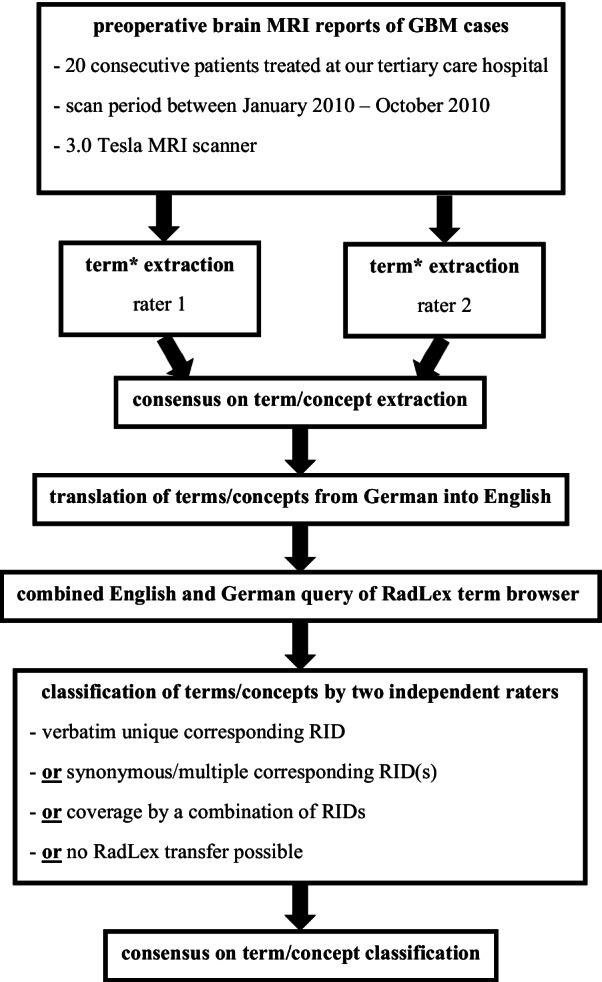
Flowchart_study protocol. This flowchart schematically visualizes the principal course of the study from top to bottom, which is described in detail in the methods section. *Two independent neuroradiologists (rater 1 and 2) collected and classified all radiological terms/concepts included in the MRI reports’ findings sections that were either describing the tumor or its effects on surrounding brain structures. Of note, both investigators were not authors of the MRI reports. Histological confirmation of GBM diagnosis was obtained in all cases. GBM = glioblastoma. MRI = magnetic resonance imaging. RadLex = radiology lexicon developed by the Radiological Society of North America. RID = RadLex identification number

MRI reports were retrieved from the hospital’s radiology information system (NEXUS/DIS GmbH, Frankfurt am Main, Germany). Additionally, neuropathological results were collected from the electronic medical records (IXSERV, ix.mid Software Technologie GmbH, Köln, Germany). The following data were gathered by two independent raters (TH with 8 years of experience in neurosurgery and 2 years in neuroradiology; CR with 23 years of clinical practice in neuroradiology): demographic patient information including age and sex, type of surgery, neuropathological confirmation of GBM diagnosis from intraoperatively obtained tissue specimen, reporting neuroradiologists as well as all terms and concepts describing the tumors and their effects on surrounding brain tissues included in the findings section of the MRI reports. To better illustrate the matching between the terms contained in GBM MRI reports and the RadLex elements, an example case has been prepared as a supplement to this manuscript (see Additional file 1). A list of all utilized terms was compiled and a translation into English language performed. Subsequently, the RadLex Term Browser was queried twice for each item (English and German) to find verbatim equivalents represented by a unique RadLex identification number (RID) (group 1) [19]. For terms, which could not be matched with a single equivalent literal RadLex item, common clinical synonyms were also added to the database search (group 2). These elements were separated from those items with a single univocal RadLex match due to the difficulties with a view to reporting exact frequencies. In case of a negative result, we tried to combine existing RadLex entities to cover the meaning of the items unrepresented so far (group 3). The remainder of terms delineates concepts that could neither be translated directly nor via an appropriate combination of existing RadLex entities (group 4). Accordingly, each and every extracted term was categorized into one of these four prespecified groups. Moreover, the frequency of term utilization in GBM reporting (n = 20) was determined. Concepts revealing a word-for-word representation in RadLex were further investigated with respect to their first order and also subordinate RadLex categories. For this purpose, the hierarchical RadLex Tree Browser was employed. All analyses were independently performed by two neuroradiological investigators (one of both is also a board certified neurosurgeon) and incongruities regarding assignments of items were solved by consensus. Figure 1 outlines the principal course of the study.

## Results

### Patients and clinical data

The study cohort comprised of a total of 20 adult GBM patients with a mean age of 68.4 ± 10.8 years. There was a preponderance of male cases (60.0%; 12/20). The vast majority of patients underwent gross total GBM resection (80.0%; 16/20) and a minority of 15.0% (3/20) presented with symptomatic epilepsy. Detailed information on demographics, diagnoses, tumor location and size as well as surgical treatment is provided by Table 1.

**Table 1:** Demographic and clinical data

	n = 20
Age at diagnosis (years; mean ± SD)	68.4 ± 10.8
Sex (male/female)	12 (60.0%)/8 (40.0%)
diagnosis_glioblastoma	19 (95.0%)
diagnosis_gliosarcoma	1 (5.0%)
Tumor location_multifocal	6 (30.0%)
Tumor location_basal ganglia/internal capsule	1 (5.0%)
Tumor location_frontal lobe	3 (15.0%)
Tumor location_frontoparietal/central	2 (10.0%)
Tumor location_parietal	2 (10.0%)
Tumor location_temporoparietal	1 (5.0%)
Tumor location_temporal	5 (25.0%)
Tumor size (mean ± SD)^a^	10.9 ± 7.2
surgery_resection	16 (80.0%)
surgery_biopsy	4 (20.0%)
Symptomatic epilepsy	3 (15.0%)

This table presents demographic and clinical details of the analyzed patient cohort. Of note, all patients included in this study suffered from grade 4 astrocytomas

SD = standard deviation

^a^Tumor size measured in terms of maximum cross-sectional area of the contrast-enhancing portion in square centimeters

### Analysis of terms utilized in high-grade glioma MRI reporting

**Fig. 2:**
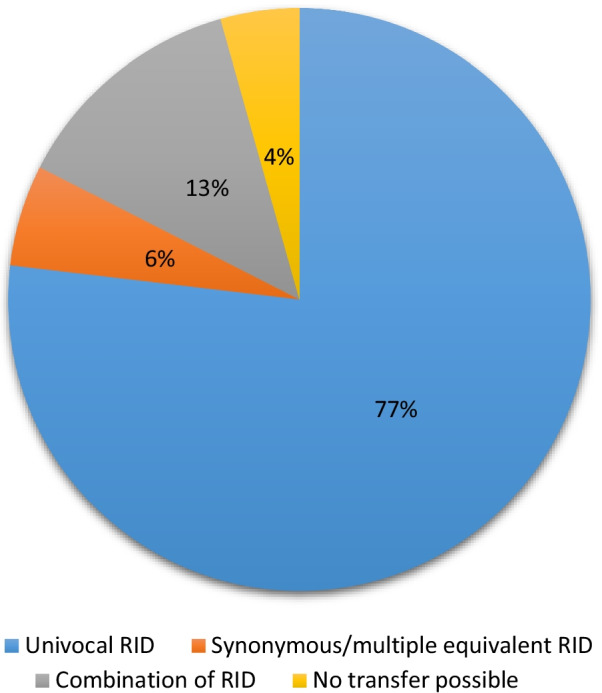
Radlex coverage of terms used in glioblastoma MRI reporting. This pie chart visualizes the broad coverage of elements used in clinical MRI reporting of glioblastomas by the RadLex terminology. RID = RadLex identification number

In-depth screening of the findings sections of the preoperative brain MRI reports on all 20 GBM patients was independently performed by two neuroradiologists and revealed a total number of 160 discrete items. The MRI reports were authored by ten experienced radiologists in total, who were not involved in the conduct of this study. 76.9% (123/160) of the extracted terms were attributable to a univocal verbatim RadLex entity. 51.2% (63/123) of these concepts with corresponding word-for-word RadLex equivalents were seen repetitively in ≥ 10% of the MRI reports. The most frequently utilized RadLex terms identified in at least half of the reports (≥ 50%) were “central” (RID 5827; 14/20 cases), “mass” (RID 3874; 12/20 cases), “necrosis” (RID 5171; 12/20 cases), “restricted diffusion” (RID 43349; 10/20 cases) and “rim enhancement” (RID 34303; 13/20 cases). A complete list of all these 123 items with verbatim RadLex matches including their German counterparts, corresponding RIDs, categorization, and prevalences in GBM MRI reporting is available as an Additional file 2 to this publication. Hierarchical classification of directly attributable RIDs into first order RadLex categories showed that most of these items fell into one of the two main categories ‘anatomical entity’ (39.0%; 48/123) or ‘RadLex descriptor’ (31.0%; 38/123), whereas the other four remaining categories together comprised of the residual 30.0% (37/123) of concepts, as presented in Table 2. Another group was set up for terms with synonymous/conceptually equivalent (i.e. non-verbatim) RadLex items and concepts with equivocal/multiple corresponding ontological RadLex entities. This group encompassed 5.6% (9/160) of all extracted elements. The most commonly encountered concepts of this cluster were ‘maximum expansion’ (3/20 cases) and ‘space occupying’ (4/20 cases). Table 3 shows all terms belonging to the second group with their respective RadLex synonyms as well as frequencies of mention in MRI reports on GBM. 13.1% (21/160) of items could only be covered by a combination of appropriate RadLex entities. ‘Perifocal edema’ (14/20 cases) and ‘facilitated diffusion’ (5/20 cases) were the most frequently employed elements in this third category. A proposal regarding RadLex transfer options and frequency of use of all these third category terms is presented in Table 4. 4.4% (7/160) of items could eventually not be transposed into existing RadLex nomenclature. Besides specific pictorial descriptions (e.g. garland-like enhancement, finger-shaped edema, areal/planar tumor spread, tumor tail), there were also other terms of infrequent usage such as ‘blood–brain barrier disruption’ and ‘main lesion’ (both concepts found in < 10% of MRI reports). In addition, RadLex offers no possibility to record an exact size measurement of a mass. The extent of a tumor could either be described by coarse segmentation into small (RID 5774), medium (RID 5775), and large (RID 5778) or alternatively less than 10 mm (RID 49805), 10–19 mm (RID 49806), and 20 mm or greater (RID 49824). Both variants lack accuracy with respect to precise characterization of the dimensions of a specific lesion. Finally, Fig. 2 graphically illustrates the extent of applicability of the RadLex terminology in glioblastoma MRI reporting.

**Table 2:** Categorization of univocal RadLex terms

RadLex main category	n = 123
Anatomical entity	48 (39.0%)
Imaging observation	19 (15.4%)
RadLex descriptor	38 (30.9%)
Clinical finding	12 (9.8%)
Property	5 (4.1%)
Procedure	1 (0.8%)

This table presents the distribution of 123 unique RadLex terms utilized for high-grade glioma MRI reporting to first order categories of the hierarchical RadLex tree

**Table 3:** Terms with synonymous or multiple equivalent RadLex matches

Term from radiology report	Equivalent Radlex terms (Radlex ID)	Frequency (n = 20)
Cella media	Body of lateral ventricle (7125)	2 (10.0%)
Contact	Adjacent (5849)	2 (10.0%)
Infiltration	Invasive (5680)/tumor invasion of adjacent structure (39,257)	2 (10.0%)
Maximum expansion	Maximum size (49,883)/diameter (13,432) + maximum (39,164)	3 (15.0%)
Narrow band	Thin rim (43,309)	1 (5.0%)
Space occupying/mass effect	Effect of mass on surrounding tissue (34,379)	4 (20.0%)
Speckled	Punctate (5900)/patchy (5704)	1 (5.0%)
Topographic relationship	Adjacent (5849)	1 (5.0%)
Weak/faint	Minor (5691)/low (46,059)	1 (5.0%)

This table shows all terms extracted from 20 consecutive glioblastoma multiforme MRI reports which have corresponding non-verbatim/synonymous or multiple equivalent RadLex entity matches

**Table 4:** Terms covered by a combination of RadLex entities

Term from radiology report	Equivalent combination of RadLex terms (RadLex ID)	Frequency (n = 20)
Facilitated diffusion	ADC value (49,527) + high (46,060)	5 (25.0%)
Frontobasal	Frontal brain region (6391) + basal surface of cerebral hemisphere (21,258)	1 (5.0%)
upper frontal region	Superolateral face of cerebral hemisphere (20,450) + frontal brain region (6391)	1 (5.0%)
Internal tumor structure	Structure (35,808) + neoplasm (3957) or lesion (38,780)	1 (5.0%)
Irregular enhancement	Irregular (5809) or heterogeneous (6060) + enhancement (34,300)	1 (5.0%)
Superomedial vertex of cerebral hemisphere^a^	Junction of body part subdivisions (33,017) + medial surface of cerebral hemisphere (21,261) + superolateral face of cerebral hemisphere (20,450)	2 (10.0%)
Midline shift	Midline (5826) + displacement (4751)	1 (5.0%)
Normal perfusion pattern	Perfusion imaging observation (38,774) + normal (13,173)	1 (5.0%)
Paramedian	Median (5846) + adjacent (5849)	1 (5.0%)
Parietobasal	Subdivision of basal surface of cerebral hemisphere (21,264) + parietal brain region (6394)	1 (5.0%)
Pericentral	Central sulcus (6456) + adjacent (5849)	1 (5.0%)
Perifocal edema	Perilesional tissue characteristics (43,362) + edema (4865)	14 (70.0%)
Peritrigonal	Collateral trigone (27,786) or collateral trigone of lateral ventricle (7135) + adjacent (5849)	2 (10.0%)
Physiologic diffusion pattern	Diffusion (10,374) + normal (13,173)	3 (15.0%)
Roof of lateral ventricle	Wall of lateral ventricle (13,822) + upper (46,057)	1 (5.0%)
Signs of malignancy	Imaging observation (5) + suggestive (39,481) + malignant (15,655)	1 (5.0%)
Subependymal	Ependyma proper (19,270) + adjacent (5849)	2 (10.0%)
Sulcal effacement	Subarachnoid space (7119) + reduced (49,912) or narrow (10,410)	2 (10.0%)
T1 hypointensity	t1 weighted (10,794) + hypointense (35,804)	1 (5.0%)
Temporobasal	Subdivision of basal surface of cerebral hemisphere (21,264) + temporal brain region (6392)	1 (5.0%)
Slight/faint enhancement	Lesion enhancement (43,365) or enhancement (34,300) + minor (5691) or low (46,059)	3 (15.0%)

This table lists all terms gathered from twenty glioblastoma MRI reports that are appropriately described by means of a combination of two or more RadLex entities

ADC = apparent diffusion coefficient

^a^Commonly used German expression ‘Mantelkante’

## Discussion

The basic goal of this feasibility study was to verify the applicability and suitability of the RadLex ontology in the reporting of gliomas, which has not been performed so far. Only under this basic condition may the benefits of a broad application of this standardized terminology in the given context be realized, namely the reduction of variation and increase of clarity in radiology reports [20]. By abstracting the inter-individual diverse vocabulary to a conceptual level in terms of specific RadLex items, comparability of findings (e.g. follow-up versus initial MRI exams) in clinical day-to-day practice as well as large-scale data aggregation for epidemiological research purposes and health care quality management measures would be facilitated because the heterogeneity and thus complexity of linguistic processing would be markedly reduced. In concrete terms, consistent application of this terminological standard in glioma reporting would make it possible to extract the language-encoded information by natural language processing software and to network it with other electronic health systems within the framework of an integrated infrastructure. This would be beneficial to brain tumor patients due to improved and less ambiguous interdisciplinary communication and additionally contribute to the development of comprehensive databases, which could be used for epidemiological research, health care decision making, and training applications. One example of such successful use for educational purposes is the integration of RadLex in medical content-based image retrieval algorithms to help radiologists make the right decisions through comparison with similar cases from an adequately annotated database [21]. As for the gains related to research, RadLex coding of the specific terms allows radiological findings to be easily translated into other languages unequivocally and without loss of content, which may facilitate transnational research collaborations and help patients in times of increased mobility. In addition, more comprehensive search results can be obtained through targeted database queries when terminology is standardized. Taken together, all these advances are only possible if the underlying terminology is applicable to the respective radiological context. It was the aim of our exploratory study to evaluate this basic requirement for the reporting of gliomas. We therefore transposed 20 consecutive free-text brain MRI reports on patients with newly diagnosed GBM into the current version of the RadLex ontology. More than 95% of descriptive terms used in these reports were satisfactorily covered by means of verbatim, synonymous, or combinations of existing RadLex items. More than a decade ago, Marwede and colleagues investigated a preliminary RadLex version with regard to indexing of thoracic computed tomography reports and observed a degree of completeness of 84% for this radiological subspecialty [9]. Furthermore, an analysis of a large set of published radiology reporting templates demonstrated a partial or complete match between 2.509 extracted unique terms and corresponding RadLex elements in a proportion of 67% [22]. A comprehensive evaluation of more than 385,000 radiology-centric figure captions gathered from 613 peer-reviewed medical journals revealed the best term mapping performance for RadLex compared to five other biomedical ontologies despite comprising fewer items [8]. Since then, the Radiological Society of North America has made huge efforts to elaborate and extend this controlled vocabulary. Therefore, the current fourth version of RadLex includes more than 46,600 distinct entities [7]. Nonetheless, there remains an inherent risk of fragmentariness of the RadLex ontology owing to its top-down construction process by expert committees with experience in various radiological subdomains [4]. Fortunately, several attempts have been undertaken in the past to overcome this structural downside by means of automatic software based extraction of terms from different large-scale sources including a corpus of Pubmed repository articles as well as an enormous set of 270,540 free-text mammography reports [10, 23]. The latter study was performed with the aid of natural language processing, which could be an option to expand other radiology domains as well by using this complementary approach. Shore and colleagues scrutinized books, radiological articles, dictionaries, and biomedical webpages for names and synonyms of imaging signs that were subsequently integrated into RadLex’s “imaging observation” section to improve its applicability [24]. Following this bottom-up concept, we manually analyzed consecutive free-text brain MRI reports of a cohort of high-grade glioma patients. The assessment displayed a small fraction of terms (< 5.0%) that could not be attributed to a specific RadLex entity or RID combination, respectively. First of all, the lexicon lacks options to communicate the exact magnitude of a specific tumorous lesion. Apart from coarse size descriptors such as small, medium, and large or alternatively less than 10 mm, 10–19 mm, and 20 mm or greater, there is no further possibility of refining this important characteristic [3]. Especially in view of patients suffering from GBM, spatial tumor dimensions are a well-established independent prognostic factor with respect to overall survival together with other clinicopathological features such as greater extent of resection, younger patient age, better physical condition, and eventually O-6-methylguanine-DNA-methyltransferase promoter methylation status [25, 26]. Bearing in mind this clinical implication, we would welcome the implementation of more detailed size descriptors in the upcoming updates on the RadLex vocabulary. Furthermore, the concept of ‘blood–brain barrier disruption’ was stated in one of the surveyed radiological documents and could not be matched adequately by any RID. On the one hand, it is not possible to visualize the blood–brain interface directly via conventional MRI—what you might see is a T1 enhancement due to extravasation of contrast medium into the extracellular space as a result of a disruption of this neuroprotective barrier, but not the barrier itself [27, 28] and therefore it could be argued that this concept may be expendable. On the other hand, a primary range of the RadLex application involves indexing of large databases of radiological free-text reports for educational and research purposes, which necessarily requires the highest achievable degree of completeness [3, 6]. Hence the issue of implementation of a specific term into RadLex should be decided upon preferably by the fact how deeply ingrained this concept is in our radiological everyday communication. A substantial part of items that were represented in the analyzed GBM MRI reports and not attributable to any specific RadLex entities could be delineated as pictorial signs, such as finger-shaped brain edema or garland-like tumor enhancement. Despite a certain lack of objectivity of such descriptions, these kinds of figurative terminological elements have been appreciated and widely accepted by radiologists for interpretations in neuroimaging right from the beginning of the era of clinical computed tomography application [29, 30]. As early as in the mid-seventies of the twentieth century a pioneer report on the diagnostic possibilities of cranial computed tomography made references to tumor-related white matter brain edema “producing finger-like shapes” in a large cohort of patients [29]. If the RadLex terminology is meant to be a common lingua franca for the radiological community, it should be oriented towards the principles of general language evolution. The Duden dictionary, first published by Konrad Duden in 1880, provides the preeminent language resource of the German language and states the authoritative rules regarding utilization of German language. It is regularly updated and the editorial decision on inclusion of a particular word or phrase is mainly based on its frequency and longevity of use [31]. Because of the widespread adoption and long-term usage of the above mentioned figurative radiological terms we propose the augmentation of the RadLex vocabulary with these elements. This approach would be well in line with the general policy pursued by the Duden curators in terms of everyday language. The synergy of a continuously expanded and updated RadLex terminology adapted to everyday practice and a set of essential morphologically describable features, as developed in the VASARI project for brain tumors by The Cancer Imaging Archive (TCIA), has the potential to sustainably improve the quality, precision, and communication of MRI reporting of GBM [32].

This study is not without limitations. The monocentric study design as well as a relatively small sample size, which was chosen due to the tremendous efforts required for manual data extraction, make up downsides of the survey potentially compromising its generalizability. On the other hand, the clearly defined eligibility criteria and thorough scrutiny of all consecutive MRI reports by two independent neuroradiological raters assure an explicit statement on the issue of the applicability of RadLex in GBM MRI reporting. Moreover, the included MRI reports were authored by a large group of ten experienced neuroradiologists all contributing their specific reporting style and vocabulary, which may increase the variability of the terms used and thus tests the basic practicability of the RadLex ontology studied in this specific neuro-oncological context.

## Conclusion

In conclusion, the thorough investigation of a consecutive set of free-text GBM MRI reports unveiled a high rate of item coverage for the RadLex terminology underscoring its representativity in this specific setting. Therefore, RadLex offers a sufficient English- and German-language tool for high-grade glioma MRI reporting that undergoes continuous further adjustment to the needs of its users, the radiological community. A precise, unambiguous, consistent, and universally applied terminology could potentially improve radiological reporting in clinical practice, thereby enhance communication with referring physicians or other medical specialists, and thus ultimately help them provide better patient care [1, 2, 33, 34]. Apart from refinement in professional communication, propagated employment of this lexicon has also the potential to advance neuroradiological education and research efforts based on data mining via natural language processing. Large-scale follow-up studies using machine learning methods are needed to confirm our results and develop these databases.

## Supplementary Information

**Additional file 1**. Example case. This example case of a typical glioblastoma magnetic resonance imaging report illustrates the applicability of the RSNA RadLex terminology in the clinical setting. The employed clinical terms are accompanied by their respective preferred RadLex names and identification numbers (RID) according to the RadLex Tree Browser (version 4.1; www.radlex.org; last accessed October 6, 2021). The related MR images of the case are provided in additionTerms with univocal RadLex ID. This table presents all radiological terms that were extracted from 20 consecutive brain magnetic resonance imaging reports on patients with newly diagnosed glioblastoma and could subsequently be attributed to a unique RadLex identification number (RID). Besides the corresponding RID, the preferred German equivalent name, RadLex categorization, and frequency of reports containing the item are provided for every term

**Additional file 2**. Terms with univocal RadLex ID. This table presents all radiological terms that were extracted from 20 consecutive brain magnetic resonance imaging reports on patients with newly diagnosed glioblastoma and could subsequently be attributed to a unique RadLex identification number (RID). Besides the corresponding RID, the preferred German equivalent name, RadLex categorization, and frequency of reports containing the item are provided for every term

## Data Availability

All data generated or analysed during this study are included in this published article and its supplementary information files.

## Abbreviations

GBM: Glioblastoma
MRI: Magnetic resonance imaging
RadLex: Radiology lexicon developed by the Radiological Society of North America
RID: RadLex identification number
