# Supplementary material for: Determining the applicability of the RSNA radiology lexicon (RadLex) in high-grade glioma MRI reporting—a preliminary study on 20 consecutive cases with newly diagnosed glioblastoma
Source: BMC Med Imaging. 2022 Mar 24;22:53. doi: 10.1186/s12880-022-00776-8 (PMC8944106; doi:10.1186/s12880-022-00776-8)
Supplement: Supplementary file 1 — Additional file 1. Example case. This example case of a typical glioblastoma magnetic resonance imaging report illustrates the applicability of the RSNA RadLex terminology in the clinical setting. The employed clinical terms are accompanied by their respective preferred RadLex names and identification numbers (RID) according to the RadLex Tree Browser (version 4.1; www.radlex.org; last accessed October 6, 2021). The related MR images of the case are provided in additionTerms with univocal RadLex ID. This table presents all radiological terms that were extracted from 20 consecutive brain magnetic resonance imaging reports on patients with newly diagnosed glioblastoma and could subsequently be attributed to a unique RadLex identification number (RID). Besides the corresponding RID, the preferred German equivalent name, RadLex categorization, and frequency of reports containing the item are provided for every term [file 12880_2022_776_MOESM1_ESM.pdf]

Additional file 1_example case

This example case of a typical glioblastoma MRI report illustrates the applicability of the RSNA RadLex terminology in the clinical setting. The employed clinical terms are accompanied by their respective preferred RadLex names and identification numbers (RID) according to the RadLex Tree Browser (version 4.1; www.radlex.org; last accessed October 6, 2021). The related MR images of the patient are provided below.

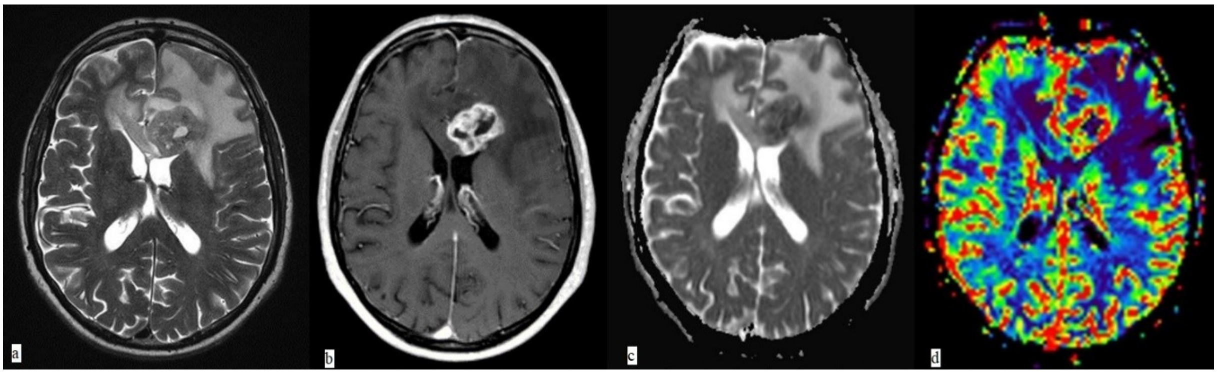

Axial MRI slices of the brain: a=T2 TSE, b=T1 MPRAGE + contrast agent (gadobutrol), c=apparent diffusion coefficient map (b-values: 0 s/mm² and 1000 s/mm²), d=relative cerebral blood volume map (dynamic susceptibility contrast MRI perfusion).

Technique:

Axial T2 TSE, sagittal T1 MPRAGE +/- contrast agent (gadobutrol) with multiplanar reconstruction, diffusion weighted imaging / apparent diffusion coefficient maps (b-values: 0 s/mm² and 1000 s/mm²), relative cerebral blood volume maps (dynamic susceptibility contrast MRI perfusion)

Findings:

Left *(left / RID 5824)* frontal *(frontal brain region / RID 6391)* irregularly shaped *(irregular / RID 5809)* lesion *(lesion / RID 38780)* adjacent *(adjacent / RID 5849)* to the frontal horn of the left lateral ventricle *(frontal horn of left lateral ventricle / RID 13799)* with *involvement (tumor invasion of adjacent structure / RID 39257)* of the genu *(genu of corpus callosum / RID 6918)* and anterior part *(anterior / RID 5818)* of the truncus of the corpus callosum *(body of corpus callosum / RID 6917)*. The mass *(mass / RID 3874)* is inhomogeneously *(heterogeneous / RID 6060)* hyperintense on T2 weighted images *(T2 hyperintensity / RID 39467)* with intratumoral *(intratumor / RID 11156)* t2 hyperintense *(T2 hyperintensity / RID 39467)* cystic *(cystic / RID 5739)* alterations *(change / RID 49896)*. Small *(small / RID 5774)* intralesional *(intralesional / RID 11134)* hyperintense *(hyperintense / RID 35805)* foci *(foci / RID 34302)* on nonenhanced t1 weighted images *(T1 weighted / RID 10794)* suggest tumor microhemorrhage *(blood products in mass / RID 43346; intratumor / RID 11156 + hemorrhage / RID 4700)*. On contrast enhanced t1 weighted images *(post-contrast / RID 28694 + T1 weighted / RID 10794)*, the lesion *(lesion / RID 38780)* shows broad *(wide / RID 39187)* irregular *(irregular / RID 5809)* rim enhancement *(rim enhancement / RID 34303)* with large *(large / RID 5778)* central *(central / RID 5827)* necrotic *(necrosis / RID 5171)* hypointense *(hypointense / RID 35804)* parts *(major part / RID 5952)*. *The adjacent (adjacent / RID 5849) ependyma (ependyma proper / RID 19270) is not affected (enhancement / RID 34300 + none / RID 28454)*. The maximum extent *(maximum size/ RID 49883)* of the contrast enhancing *(enhancing / RID 6055)* tumor *tissue (neoplasm / RID 3957 + portion of*

tissue / RID 13298) is 3.6 cm x 2.7 cm ***(no accurate size descriptor available; ≥20mm / RID 49824)*** on axial planes *(axial plane / RID 10579)*. T2-hyperintense signal alterations *(T2 hyperintensity / RID 39467)* are extensive *(extensive / RID 5685)* and embrace *(tumor invasion of adjacent structure / RID 39257)* the left (*(left / RID 5824)* superior *(left superior frontal gyrus / RID 13903)*, middle *(left middle frontal gyrus / RID 13908),* and inferior frontal gyri *(left inferior frontal gyrus / RID 6979)* as well as the left external capsule *(left external capsule / RID 17041)* and extend through the genu of the corpus callosum *(genu of corpus callosum / RID 6918)* to the contralateral *(contralateral / RID 39254*) periventricular *(periventricular / RID 6384)* white matter *(segment of cerebral white matter / RID 16999)*. The tumor *(neoplasm / RID 3957)* and its related edema (edema / RID 4865) cause mild (mild / RID 5671) midline (midline / RID 5826; interhemispheric fissure / RID 6557) shift to the right side *(displacement / RID 4751 + right / RID 5825)* and severe *(severe / RID 5673)* compression *(compression / RID 4741)* of the frontal horn of the left lateral ventricle *(frontal horn of left lateral ventricle / RID 13799)*. Parts of the tumor present with restricted diffusion *(partial / RID 5687 + intratumor / RID 11156 + restricted diffusion / RID 43349)* on the apparent diffusion coefficient map *(apparent diffusion coefficient map / RID 12698)*. The enhancing *(enhancing / RID 6055)* part of the mass (mass / RID 3874) displays extensive *(extensive / RID 5685)* hyperperfusion *(hyperperfusion / RID 4978)* on the relative cerebral blood volume map *(cerebral blood volume map / RID 28489)*.
