# Supplementary material for: Determining the applicability of the RSNA radiology lexicon (RadLex) in high-grade glioma MRI reporting—a preliminary study on 20 consecutive cases with newly diagnosed glioblastoma
Source: BMC Med Imaging. 2022 Mar 24;22:53. doi: 10.1186/s12880-022-00776-8 (PMC8944106; doi:10.1186/s12880-022-00776-8)
Supplement: Supplementary file 2 — Additional file 2. Terms with univocal RadLex ID. This table presents all radiological terms that were extracted from 20 consecutive brain magnetic resonance imaging reports on patients with newly diagnosed glioblastoma and could subsequently be attributed to a unique RadLex identification number (RID). Besides the corresponding RID, the preferred German equivalent name, RadLex categorization, and frequency of reports containing the item are provided for every term [file 12880_2022_776_MOESM2_ESM.pdf]

terms with corresponding univocal RID	preferred German name	RID	RadLex subcategory (RadLex first order category)	frequency (n=20)
adjacent	angrenzend	5849	location descriptor (radlex descriptor)	4
anterior limb of internal capsule	crus anterius capsulae internae	6942	subdivision of internal capsule (anatomical entity)	1
anatomical junction	anatomische Aufzweigung	13231	anatomical cluster (anatomical entity)	1
anterior	anterior	5818	location descriptor (radlex descriptor)	1
attachment	Anhaftung	46017	visible anatomic entity (imaging observation)	1
basal ganglion of telencephalon	Basalganglien	15697	ganglion of neuraxis (anatomical entity)	1
basal surface of cerebral hemisphere	basale Oberfläche der Hirnhemisphäre	21258	subdivision of surface of cerebral hemisphere (anatomical entity)	1
body of lateral ventricle	ventriculus lateralis, pars centralis	7125	subdivision of lateral ventricle (anatomical entity)	1
calcification	Kalzifikation	5196	mineral deposition disorder (clinical finding)	1
caudal	kaudal	5832	location descriptor (radlex descriptor)	2
central	zentral	5827	location descriptor (radlex descriptor)	14
cerebral hemisphere	Großhirnhemisphäre	13857	segment of forebrain (anatomical entity)	1
cerebral white matter	weiße Substanz des Cerebrums	16996	white matter of telencephalon (anatomical entity)	1
choroid plexus of lateral ventricle	plexus choroideus ventriculi lateralis	7132	region of wall of lateral ventricle (anatomical entity)	1
cingulate gyrus	gyrus cinguli	6510	gyrus of limbic lobe (anatomical entity)	1
circumferential enhancement	Randenhancement	6061	enhancement pattern (imaging observation)	2
circumscribed margin	umschriebener Rand	5707	margin (imaging observation)	1
collateral trigone	trigonum collaterale	27786	region of wall of lateral ventricle (anatomical entity)	1
collateral trigone of lateral ventricle	trigonum collaterale des Seitenventrikels	7135	subdivision of lateral ventricle (anatomical entity)	1
compression	Kompression	4741	architectural distortion (clinical finding)	1
contiguous	angrenzend	39044	orientation descriptor (radlex descriptor)	1
contralateral	kontralateral	39254	location descriptor (radlex descriptor)	1
coronal	koronar	5861	orientation descriptor (radlex descriptor)	1
cranial	kranial	5831	anatomically-related descriptor (radlex descriptor)	1
craniocaudal	kraniokaudal	49908	anatomically-related descriptor (radlex descriptor)	3
cystic	zystisch	5739	composition descriptor (radlex descriptor)	1
diameter	Durchmesser	13432	measurement type (property)	3
diffusion	Diffusion	10374	imaging procedure (procedure)	1
disease spread	Erkrankungsausbreitung	5230	pathophysiologic finding (clinical finding)	1
displacement	Verlagerung	4751	mechanical disorder (clinical finding)	3
distension	Dehnung	3793	enlargement (clinical finding)	1
dorsal	dorsal	5847	anatomically-related descriptor (radlex descriptor)	4
dura/dura mater	dura mater	7093	membrane organ (anatomical entity)	2
edema	Ödem	4865	fluid disorder (clinical finding)	4
enhancement (syn: area of enhancement)	Verstärkung (Syn: Anreicherung)	34300	imaging observation (imaging observation)	3
enhancing	anreichernd	6055	enhancement pattern (imaging observation)	3
extensive	ausgedehnt	5685	status descriptor (radlex descriptor)	7
falx cerebri	falx cerebri	7094	subdivision of cranial dura mater (anatomical entity)	2
fluid-attenuated inversion recovery	Fluid Attenuated Inversion Recovery (FLAIR) (Liquorsuppression)	35806	MR tissue contrast attribute (property)	1
focal enhancement	fokale Verstärkung (Syn: fokale Anreicherung)	34335	non-mass enhancement distribution (imaging observation)	2
frontal brain region	frontale Hirnregion	6391	brain region (anatomical entity)	4
frontal horn of lateral ventricle	cornu anterius ventriculi lateralis	7126	subdivision of lateral ventricle (anatomical entity)	2
frontal operculum	operculum frontale	20410	zone of telencephalon (anatomical entity)	1
frontoparietal brain region	fronto-parietale Hirnregion	6395	brain region (anatomical entity)	2
genu of corpus callosum	genu corporis callosi	6918	segment of corpus callosum (anatomical entity)	2
growth	Wachstum	39547	property (property)	1
hemispheric	hemisperiell	6402	head pathophysiologic process observation descriptor (radlex descriptor)	1
hemorrhage	Hämorrhagie	4700	flow disorder (clinical finding)	2
heterogeneous (syn: inhomogeneous)	heterogen (Syn: inhomogen)	6060	uniformity descriptor (radlex descriptor)	5
heterogeneous enhancement	heterogene Signalverstärkung	39457	enhancement uniformity (imaging observation)	1
high signal intensity	hohe Signalintensität	6052	signal characteristic (radlex descriptor)	1
hippocampus	Hippocampus	6529	gyrus of limbic lobe (anatomical entity)	1
homogeneous enhancement	homogene Signalverstärkung	39563	enhancement uniformity (imaging observation)	1
hyperintense	hyperintens	35805	signal characteristic (radlex descriptor)	2
hyperperfusion	Hyperperfusion	4978	perfusion disorder (clinical finding)	6
hypervascular lesion	hypervaskuläre Läsion	34389	lesion (imaging observation)	5
hypointense	hypointens	35804	signal characteristic (radlex descriptor)	2
indistinct margin	unscharfer Rand	5709	non-circumscribed margin (imaging observation)	3
inferior frontal gyrus	gyrus frontalis inferior	6443	gyrus of frontal lobe (anatomical entity)	1
inferior occipital gyrus	gyrus occipitalis inferior	26548	gyrus of occipital lobe (anatomical entity)	1
inferior temporal gyrus	gyrus temporalis inferior	6479	gyrus of temporal lobe (anatomical entity)	1
insula	lobus insularis	6472	lobe of cerebral hemisphere (anatomical entity)	1
interhemispheric fissure	Interhemisphärenspalt	6557	subarachnoid fissure (anatomical entity)	2
internal capsule	capsula interna	6941	capsule of cerebral hemisphere (anatomical entity)	1
invasive	invasiv	5680	aggressiveness descriptor (radlex descriptor)	1
large	groß	5778	size descriptor (radlex descriptor)	2
lateral	lateral	39121	location descriptor (radlex descriptor)	1
lateral ventricle	lateraler Ventrikel	7124	cerebral ventricle (anatomical entity)	2
lesion	Läsion	38780	enhancement (imaging observation)	3
malignant neoplasm	maligne Neoplasie	36042	neoplasm (clinical finding)	1
marked	deutlich (Syn: ausgeprägt)	34299	status descriptor (radlex descriptor)	2
mass	Raumforderung	3874	lesion (imaging observation)	12
maximum size	-	49883	measurement type (property)	2
medial	medial	5820	location descriptor (radlex descriptor)	2
midbrain	mesencephalon	6768	cardinal segment of brain (anatomical entity)	1
middle temporal gyrus	gyrus temporalis medius	6478	gyrus of temporal lobe (anatomical entity)	2
midline	Mittellinie	5826	location descriptor (radlex descriptor)	2
mottled enhancement	gesprenkeltes Muster der Signalverstärkung	43321	spatial enhancement pattern (imaging observation)	1
multifocal	multifokal	5703	distribution pattern (radlex descriptor)	1
multilocular	multilokulär	5891	morphologic descriptor (radlex descriptor)	1
multiple	multipel	5765	quantity descriptor (radlex descriptor)	1
necrosis	Nekrose	5171	degenerative disorder (clinical finding)	12
neoplasm	Neoplasie	3957	proliferation (clinical finding)	3
no	kein (Syn: nicht)	28475	certainty descriptor (radlex descriptor)	1
no effect of mass on surrounding tissue	kein Effekt einer Masse auf umliegendes Gewebe	34380	effect of mass on surrounding tissue (imaging observation)	2
nonenhancing	nicht anreichernd	6056	enhancement pattern (imaging observation)	3
occipital brain region	okzipital	6393	brain region (anatomical entity)	2
occipital horn of lateral ventricle	cornu occipitale ventriculi lateralis	7127	subdivision of lateral ventricle (anatomical entity)	2
parahippocampal gyrus	gyrus hippocampi (Syn: gyrus parahippocampi)	6531	gyrus of limbic lobe (anatomical entity)	1
parietal brain region	parietale Hirnregion	6394	brain region (anatomical entity)	1
parietal lobe	lobus parietalis	6493	lobe of cerebral hemisphere (anatomical entity)	1
patchy	fleckig	5704	distribution pattern (radlex descriptor)	1
peripheral	peripher	5828	location descriptor (radlex descriptor)	2
periventricular	periventrikulär	6384	head location descriptor (radlex descriptor)	1
petrous part of temporal bone	pars petrosa des os temporale	9362	zone of temporal bone (anatomical entity)	1
planum temporale	planum temporale	34220	caviness neocortical parcellation unit (anatomical entity)	1
postcentral gyrus	gyrus centralis posterior (Syn: gyrus postcentralis)	6494	gyrus of parietal lobe (anatomical entity)	3
precentral gyrus	gyrus precentralis	6448	gyrus of frontal lobe (anatomical entity)	5
progressive	progressiv	39162	status descriptor (radlex descriptor)	1
pyramidal tract	Pyramidenbahn	17623	tract of brain (anatomical entity)	2
restricted diffusion	eingeschränkte Diffusion	43349	enhancement pattern (imaging observation)	10
rim	Rand	5980	anatomic sub-part (anatomical entity)	2
rim enhancement	Signalverstärkung des Rands	34303	mass internal enhancement pattern (imaging observation)	13
round	rund	5799	morphologic descriptor (radlex descriptor)	2
sagittal	sagittal	5860	orientation descriptor (radlex descriptor)	1
satellite lesions present	Satellitenläsionen vorhanden	43317	lesion load (imaging observation)	5
semioval center	centrum semiovale	6959	radiation of cerebral hemisphere (anatomical entity)	2
small	klein	5774	size descriptor (radlex descriptor)	3
smooth margin	glatter Rand	5714	circumscribed margin (imaging observation)	1
solid	solide	5741	solidness descriptor (radlex descriptor)	1
splenium of corpus callosum	corpus callosum splenium (Syn: splenium corporis callosi)	6916	segment of corpus callosum (anatomical entity)	3
subcortical	subkortikal	6387	head location descriptor (radlex descriptor)	2
superior occipital gyrus	gyrus occipitalis superior	13933	gyrus of occipital lobe (anatomical entity)	1
superior temporal gyrus	gyrus temporalis superior	6477	gyrus of temporal lobe (anatomical entity)	2
t1 weighted	T1 gewichtet	10794	MR tissue contrast attribute (property)	2
t2 hyperintensity	T2-Hyperintensität	39467	hyperintense (radlex descriptor)	4
t2 hypointensity	T2-Hypointensität	49501	hypointense (radlex descriptor)	1
temporal brain region	temporale Hirnregion	6392	brain region (anatomical entity)	3
temporal horn of lateral ventricle	cornu inferius ventriculi lateralis (Syn: cornu temporale ventriculi lateralis)	7128	subdivision of lateral ventricle (anatomical entity)	2
temporal lobe	lobus temporalis	6476	lobe of cerebral hemisphere (anatomical entity)	3
temporal pole	polus temporalis	20414	zone of telencephalon (anatomical entity)	2
thalamus	dorsaler Thalamus	6578	organ component of neuraxis (anatomical entity)	2
torsion	Torsion	4813	rotation disorder (clinical finding)	1

Additional file 2_terms with univocal RadLex ID. This table presents all radiological terms that were extracted from 20 consecutive brain magnetic resonance imaging reports on patients with newly diagnosed glioblastoma and could subsequently be attributed to a unique RadLex identification number (RID). Besides the corresponding RID, the preferred German equivalent name, RadLex categorization, and frequency of reports containing the specific item are also provided for every term.
